# Magnetic Resonance Imaging Features of Extradural Spinal Neoplasia in 60 Dogs and Seven Cats

**DOI:** 10.3389/fvets.2020.610490

**Published:** 2021-01-07

**Authors:** Mylène Auger, Silke Hecht, Cary M. Springer

**Affiliations:** ^1^Animages, Longueuil, QC, Canada; ^2^Department of Small Animal Clinical Sciences, College of Veterinary Medicine, University of Tennessee, Knoxville, TN, United States; ^3^Research Computing Support, University of Tennessee, Knoxville, TN, United States

**Keywords:** MRI, canine, feline, spinal tumor, sarcoma, lymphoma, carcinoma, multiple myeloma

## Abstract

This retrospective study describes the MRI features of extradural spinal neoplasia in 60 dogs and seven cats to identify potential distinguishing features between tumor classes and individual tumor types within each class. In dogs, mesenchymal tumors were most common (48%), with undifferentiated sarcomas being the predominant tumor type. Round cell neoplasms were second most common (35%), with lymphoma and multiple myeloma/plasma cell tumor comprising the majority of cases. Only two benign tumors were identified. In cats, lymphoma was most common (5/7), with one case of mesenchymal neoplasia and one case of metastatic carcinoma. Despite some overlap, certain imaging features were able to help prioritize differential diagnoses. The combined features that predicted round cell neoplasia (84%) included the preservation of vertebral shape, homogeneous contrast enhancement, and lesion centering on bone. The combined features that predicted mesenchymal neoplasia (73%) included altered vertebral shape, heterogeneous contrast enhancement, and lesion centering on paraspinal soft tissues. Round cell neoplasms were more likely to have cortical sparing, preservation of overall shape, lesion centering on bone, small soft tissue tumor size, and homogeneous contrast enhancement. Both epithelial and mesenchymal neoplasms were more likely to have cortical lysis, a cavitary component to the soft tissue mass and medium to large soft tissue mass size. The findings of this study can aid in prioritizing differential diagnoses in cases of extradural spinal neoplasia in cats and dogs, which can impact case management, but tissue sampling remains the gold standard for definitive diagnosis.

## Introduction

Spinal tumors are benign or malignant proliferation of cells that can result in compression or invasion of the spinal cord, meninges, paraspinal soft tissues, and/or nerve roots and can be associated with clinical signs of spinal cord dysfunction and pain (1, 2). Spinal tumors can be primary, arising from the spinal, dural, or paraspinal tissues, or secondary, following metastasis from other locations (1, 2). Based on the anatomic location, spinal neoplasia can also be classified as extradural, intradural-extramedullary, or intramedullary (2).

Extradural tumors are the most common spinal neoplasia in cats and dogs and include primary bone neoplasms (e.g., osteosarcoma, fibrosarcoma, chondrosarcoma, and hemangiosarcoma), round cell neoplasms (e.g., multiple myeloma, lymphoma, and plasma cell tumors), primary tumors arising from paraspinal soft tissues (e.g., liposarcoma, mast cell tumor, and myxosarcoma), and metastasis, commonly carcinomas (2–6). Less commonly, benign tumors, such as osteoma, fibroma, chondroma, lipoma, and synovial myxoma, can occur (2, 7–17). Locally invasive, slow-growing neoplasms arising from remnants of the notochord, chondroid chordomas, have also rarely been reported (18, 19). Benign tumor-like proliferation of bone and connective tissues, such as with multiple cartilaginous exostosis and calcinosis circumscripta, have also occasionally been reported to affect the spine (20–22). Finally, peripheral nerve sheath tumors can occur in extradural, intradural-extramedullary, and intramedullary locations (2).

With the increasing use of cross-sectional imaging modalities such as computed tomography and magnetic resonance imaging (MRI), extradural spinal neoplasia is being increasingly recognized and treated (2). Identification of the origin and extent of a tumor is important for surgical planning and/or radiation therapy planning (1). Choice of treatment will vary based on tumor type, with surgical resection in conjunction with chemotherapy and/or radiation therapy described for primary bone tumors and spinal lymphoma, whereas radiation therapy and/or chemotherapy has been described in the treatment of plasma cell tumor and multiple myeloma (1, 2, 23–25). Prognosis depends on the extent of local resection and spinal infiltration, spinal cord damage before and during surgery, the surgeon's experience level, and tumor type (1). Multiple individual case reports and small case series have described the imaging features of various types of spinal neoplasia in cats and dogs, often combining extradural, intradural-extramedullary, and intramedullary tumors; however, a large case series comparing the imaging features of extradural tumors with confirmed diagnoses is currently lacking (1, 26–32).

Previously reported MRI findings in dogs with spinal lymphoma include vertebral involvement, with vertebral changes confined to the medullary cavity without cortical lysis, multifocal disease, and extradural spinal cord compression, although cortical lysis was described in one dog of a different report (23, 33). In the latter case, the osseous anatomy of the affected vertebra remained largely preserved (23). In cats, extradural lymphoma has been reported to consist of an epidural mass resulting in spinal cord compression, which can extend into and through the adjacent vertebrae as well as into the underlying muscle and has been reported to be T2-hyperintense to white matter, T1-hypointense, with a variable mass effect and homogeneous contrast enhancement (34, 35). An epidural mass without bone involvement has also been reported in dogs with spinal lymphoma (33). The reported MRI findings in dogs with multiple myeloma include multiple expansile vertebral lesions that do not extend beyond the vertebral outer cortical limits with the presence of extradural material, causing spinal cord compression. In contrast, mesenchymal tumors have been reported to cause more marked vertebral lysis. In fact, a previous report in a dog with vertebral osteosarcoma described marked osteolysis and a large soft tissue mass component, whereas a case series evaluating MRI findings in dogs with chondrosarcoma described mixed osteolytic and proliferative masses, which were T2-hyperintense, T1-hypointense, and involved the dorsal compartments of several consecutive vertebrae (36).

The goals of the following retrospective analytical study were to describe the MRI features of extradural neoplasia in cats and dogs and identify potential distinguishing features between tumor classes and types, which could facilitate prioritization of differential diagnoses. We hypothesized that there would be an overlap in some MRI features between tumor classes and types, particularly those pertaining to signal intensity, the involvement of more than one spinal compartment, and the degree of spinal cord compression. We also hypothesized that certain features, such as cortical sparing, preserved vertebral shape, and small soft tissue mass size would be more common with round cell tumors, whereas cortical lysis, large soft tissue tumor size, and alteration of vertebral shape would be more common with mesenchymal tumors. We predicted that lymphoma would be the most common extradural tumor in cats and that the MRI features of cats with spinal lymphoma would be similar to dogs.

## Materials and Methods

All MRI studies of the spine and their corresponding report (redacted by a board-certified veterinary radiologist who had evaluated the case at the time) performed at our institution between April 2008 and April 2020 were retrospectively reviewed. All reports describing an extradural spinal mass were retrieved from the picture archival and communication system (PACS) and reviewed by one of the investigators (MA) to confirm extradural location. Of these, cases with a diagnosis confirmed by cytology, histopathology (either from a surgical biopsy or necropsy), or using previously published diagnostic criteria (such as for multiple myeloma) were initially included (37–39). Cases with a diagnosis of undifferentiated malignant neoplasia were excluded if additional testing further narrowing the tumor type to at least a specific category was not available. Cases were also excluded if results of cytology or histopathology were inconclusive or if the diagnosis included multiple possible tumor classes without additional testing to further narrow the diagnosis.

A board-certified veterinary radiologist (MA) reviewed the medical records and retrieved the following information: signalment, results of complete blood count and blood chemistry, and results of cytology, histopathology, and bone marrow aspirate findings. Cases were categorized by tumor class (mesenchymal tumors, round cell neoplasia, epithelial neoplasia, neuroendocrine tumors, and benign tumors). When possible, cases were further subclassified into specific tumor types within their respective class.

### MRI Study Acquisition and Evaluation

MRI studies were performed using a 1-Tesla magnet (MAGNETOM Harmony^TM^, Siemens Medical Solutions, Malvern, PA) from April 2008 to January 2013 and a 1.5-Tesla magnet (MAGNETOM Espree^TM^, Siemens Medical Solutions, Malvern, PA) from February 2013 to April 2020. All MRI studies were performed under general anesthesia with the patient in dorsal recumbency. The site of imaging varied based on clinical examination findings and neurolocalization. MRI protocols varied between patients; however, all MRI studies included T2-weighted (T2W) and T1-weighted (T1W) sequences in sagittal and transverse planes and T1W images acquired after intravenous administration of gadolinium-based contrast medium (gadopentetate meglumine, Magnevist®, Bayer Schering Pharma, 0.1 mmol/kg). When available, short tau inversion recovery (STIR) and T2^*^-weighted gradient recalled echo images were also reviewed.

All MRI studies were independently reviewed by two board-certified veterinary radiologists (MA and SH) who were blinded to the final diagnosis at the time of image evaluation. Images were evaluated using a PACS workstation DICOM viewer (SECTRA PACS IDS7^TM^, Sectra Medical Systems AB, Linkoeping, Sweden) ~4 months after the initial medical records review. In cases of discrepancies between observers in image evaluation, a consensus agreement was reached.

Lesion location was determined in relation to vertebral body number and was then categorized into vertebral regions as C1-C5, C6-T2, T3-L3, and L4-S3. If multiple regions were affected, these were documented. Lesion centering was categorized as epidural soft tissues, paraspinal soft tissues, or bone.

Lesions were categorized as segmental or multifocal, where a segmental lesion was defined as a single abnormality, even if spanning multiple vertebrae, and multifocal lesions were defined as separate abnormalities attributed to the same disease process.

Invasive behavior was characterized by the presence or absence of contrast enhancement or mass extension into the paraspinal soft tissues, presence or absence of vertebral bone involvement, and presence or absence of mass extension into the vertebral canal.

Lesions were defined as monostotic or polyostotic and characterized as monostotic if only one vertebra was involved and as polyostotic when the involvement of multiple vertebrae and/or additional bones was identified. Involvement of bones other than vertebrae was also categorized as present or absent.

When the vertebral bone was involved, the following features were categorized as present or absent: cortical lysis, cortical sparing, benign or reactive vertebral changes, preservation of overall vertebral shape (excluding pathologic fractures), and pathologic fractures. Cortical lysis was defined as discontinuity of the normal T1/T2 hypointense cortical margin. When there was an increase or decrease in T2, STIR, and/or T1 signal intensity relative to adjacent vertebrae, which was limited to the medullary cavity, this was characterized as cortical sparing. Benign/reactive changes included pressure atrophy/foraminal widening, smooth periosteal proliferation, and smooth expansile bone enlargement without cortical lysis. Preservation of the overall architecture of the vertebra despite small foci of cortical lysis was characterized as preserved overall vertebral shape. In lesions with expansile lysis or with extensive areas of osteolysis disrupting osseous anatomy, this was characterized as an alteration of the overall shape.

The presence or absence of periosteal reaction was also noted. When periosteal proliferation was present, it was further classified as aggressive, when columnar, spiculated or amorphous, or benign, if smooth.

The soft tissue mass size was measured in relation to vertebral body diameter on transverse images and characterized as small (<1), medium (>1 and <2), or large (>2). Soft tissue masses were evaluated for the presence of mineralization, and this was characterized as present or absent.

The degree of spinal cord compression was classified based on the percentage of vertebral canal diameter occupied by extradural material (mild, <25%; moderate, 25–50%; severe, >50%).

Signal intensity was recorded on T2W, T1W, and STIR sequences. The signal intensity on T1W, T2W, and STIR sequences was defined as the relative signal intensity compared with the same unaffected tissue type. The paraspinal portion of the lesion was compared with the paraspinal muscles, the vertebral portion to adjacent bone, and the vertebral canal portion to the spinal cord. Signal homogeneity/heterogeneity was recorded on T2W and T1W pre-contrast sequences and subjectively defined as homogeneous, heterogeneous, or markedly heterogeneous. The presence of absence of a susceptibility artifact on T2^*^-weighted gradient recalled echo sequences (if available) was also recorded.

The presence or absence of contrast enhancement, the degree of contrast enhancement, and contrast enhancement type were recorded. The degree of contrast enhancement was subjectively defined as none, mild, moderate, and marked when compared with pre-contrast sequences and adjacent unaffected structures. Contrast enhancement type was subjectively defined as heterogeneous or homogeneous. In cases of disagreement pertaining to contrast enhancement degree or type, a consensus between observers was obtained. The presence or absence of ring enhancement and presence or absence of a cavitary component to the soft tissue mass were also recorded. A cavitary component to the soft tissue mass was characterized by non-contrast-enhancing, T2-strongly hyperintense and T1-hypointense areas within the mass.

Additional recorded features included the presence or absence of meningeal contrast enhancement, nerve root involvement, intradural extension, and other spinal cord changes such as T2 intramedullary hyperintensity and/or swelling. When a lesion followed the course of a nerve root, this was characterized as the presence of nerve root involvement. Lesion extension from the vertebral canal into an intervertebral foramen but not beyond was not considered consistent with nerve root involvement.

Evaluators were also asked to note the presence of likely clinically relevant lesions outside of the vertebral column included in the study, such as peripheral, thoracic, or abdominal lymph node involvement, thoracic or abdominal masses or nodules, generalized organomegaly, cutaneous/subcutaneous/muscle nodules and/or masses, muscle atrophy, and pleural or peritoneal effusion.

### Statistical Analysis

Statistical analysis was performed by a statistician (C.M.S.), and all analyses were conducted using commercially available statistics software (IBM SPSS Statistics for Windows, version 26, IBM Corp., Armonk, NY, USA). Chi-square analysis was performed to evaluate possible relationships between tumor classes and imaging features and between tumor types and imaging features. Binary logistic regression was performed to identify individual features or combinations of features that could predict tumor class. For statistical evaluation of tumor classes, mesenchymal tumors, round cell tumors, and epithelial tumors were included in the statistical analysis. Tumors from the neuroendocrine and benign classes were excluded from statistical analysis due to low case numbers. For statistical evaluation of specific tumor types on MRI, in the round cell tumor class, only lymphoma and multiple myeloma/plasma cell tumor were included due to low count numbers in the other tumor type categories. Given the similarities in imaging findings with lymphoma in between cats and dogs, these were grouped together for statistical analysis. In the mesenchymal tumor class, osteosarcoma, chondrosarcoma, and hemangiosarcoma were grouped together for statistical analysis of specific tumor types (a group named “primary bone tumors”) and compared with peripheral nerve sheath tumors (PNSTs) and undifferentiated sarcomas. Soft tissue sarcomas were excluded from statistical analysis of tumor types due to low case numbers. For evaluation of specific imaging features, if both cortical lysis and cortical sparing were seen in the same study, the most aggressive feature (cortical lysis) was used for statistical analysis. If multiple soft tissue masses were present, the largest was used for statistical analysis. With multifocal sites of spinal cord compression, the most severe site was used for statistical analysis. Significance was set at *p* < 0.05.

## Results

### Study Population

A total of 70 patients were initially included. Of these, two dogs and one cat were excluded because results of cytology and/or histopathology were consistent with undifferentiated malignant neoplasia, and further classification into at least a specific tumor class was not available. A total of 67 patients were therefore included, of which 60 were dogs and seven were cats.

Affected canine breeds were 23 mixed breed dogs, seven Labrador Retrievers, three Boxers, three Yorkshire Terriers, two German Shepherd dogs, two Dachshunds, two Miniature Schnauzers, and one of each Akita, Australian Cattle Dog, Australian Shepherd, Beagle, Boston Terrier, Bullmastiff, Cavalier King Charles Spaniel, Cocker Spaniel, English Setter, Golden Retriever, Great Dane, Greyhound, Maltese, Otterhound, Rottweiler, Shar Pei, Standard Schnauzer, and Toy Poodle. Ages ranged between 5 months and 13 years of age, with a median age of 9 years old and a mean age of 8.8 years of age ±3.0 years. Twenty-eight dogs were spayed females, four dogs were intact males, and 28 dogs were castrated males.

All included cats were domestic shorthair cats and ranged between 1 and 20 years of age with a median age of 10 years old and a mean age of 10.1 ± 6 years. One cat was a spayed female, whereas the remaining six cats were castrated males.

### Image Acquisition MRI

Thirty-three MRI studies were performed using a 1-Tesla magnet and 34 using a 1.5-Tesla magnet.

### Diagnosis

The diagnoses were obtained by necropsy/histopathology in 22/67 (32.8%) of cases, by cytology of the extradural lesion in 18/67 (26.9%) of cases, by surgical biopsy and histopathology of the lesion in 16/67 (23.9%) of cases, by cytology of other abdominal, thoracic, musculoskeletal, and/or lymph node lesions in cases of disseminated disease in 9/67 (13.4%) of cases, by surgical biopsy of other lesions in cases of disseminated disease in 1/67 (1.5%) of cases, and by using previously published criteria for the diagnosis of multiple myeloma in 1/67 (1.5%) of cases.

In dogs, due to the multifocal nature of lesions in 17 cases, a total of 79 spinal regions (C1-C5, C6-T2, T3-L3, and L4-S3) were affected in 60 dogs. The most commonly affected spinal region was at the level of the T3-L3 vertebrae (36/79), followed by L4-S3 (20/79), C1-C5 (12/79), and C6-T2 (11/79). In 15 cases, lesions were seen in two regions: T3-L3 and L4-S3 in 10/15, C1-C5 and C6-T2 in 3/15, and C6-T2 and T3-L3 in 2/15. In two cases, lesions were seen in three regions, C6-T2, T3-L3, and L4-S3.

In cats, due to the multifocal nature of lesions in two cases, a total of nine spinal regions (C1-C5, C6-T2, T3-L3, and L4-S3) were affected in seven cats. The most commonly affected spinal region was at the level of the L4-S3 vertebrae (5/9), followed by T3-L3 (3/9), and C1-C5 (1/9). In two cases, lesions were seen in two regions, T3-L3 and L4-S3.

In dogs, mesenchymal tumors were diagnosed in 29/60 (48.3%), round cell neoplasia in 21/60 (35%), epithelial neoplasia in 7/60 (11.7%), neuroendocrine tumors in 1/60 (1.7%), and benign tumor types/mass lesions in 2/60 (3.3%). Mesenchymal tumor types included undifferentiated sarcoma (11/29), osteosarcoma (5/29), PNST (6/29), chondrosarcoma (3/29), hemangiosarcoma (2/29), and soft tissue sarcoma (2/29). Round cell tumor types included lymphoma (7/21), multiple myeloma/plasma cell tumor (7/21), histiocytic sarcoma (3/21), and round cell neoplasia of undetermined cell type (4/21). Epithelial tumor types included thyroid carcinoma (3/7) and metastatic carcinoma (4/7), with specific metastatic tumor types consisting of prostatic carcinoma (2/4), hepatic cholangiocarcinoma (1/4), and apocrine gland adenocarcinoma of the anal sac (1/4). Neuroendocrine tumor types consisted of a carotid body tumor (1/1). Benign tumor/mass-lesion types included calcinosis circumscripta (1/2) and synovial myxoma (1/2).

In cats, round cell neoplasia was diagnosed in 5/7 (71%), mesenchymal tumors were diagnosed in 1/7 (14%), and epithelial neoplasia in 1/7 (14%). Round cell tumor types consisted of lymphoma in 5/5 (100%). Epithelial neoplasia tumor types consisted of metastatic intestinal carcinoma in 1/1 (100%). Mesenchymal neoplasia tumor types consisted of a PNST in 1/1 (100%).

### MRI Features Based on Tumor Classes

Table 1 summarizes the tumor classes and types seen with MRI in this study.

**Table 1:** Summary of the tumor classes and individual tumor types seen in the dogs and cats of this study.

**Class**		**Type**	**Frequency**	**Percent of class**	**Percent of types**
Mesenchymal tumors		Undifferentiated sarcoma	11	36.7	16.4
		Peripheral nerve sheath tumor	7	23.3	10.4
		Osteosarcoma	5	16.7	7.4
		Chondrosarcoma	3	10	4.5
		Hemangiosarcoma	2	6.7	3
		Soft tissue sarcoma	2	6.7	3
	**Class total**		**30**	**100**	**44.8**
Round cell tumors		Lymphoma	12	46.2	17.9
		Multiple myeloma/plasma cell tumor	7	26.9	10.4
		Undetermined tumor type	4	15.4	6
		Histiocytic sarcoma	3	11.5	4.5
	**Class total**		**26**	**100**	**38.8**
Epithelial tumors		Metastatic carcinoma	5	62.5	7.5
		Thyroid carcinoma	3	37.5	4.5
	**Class total**		**8**	**100**	**11.9**
Neuroendocrine tumors		Carotid body tumor	1	100	1.5
	**Class total**		**1**	**100**	**1.5**
	**Overall total**		**67**		**100**
Benign		Calcinosis circumscripta	1	50	1.5
		Synovial myxoma	1	50	1.5
	**Class total**		**2**	**100**	**3**

**Figure 1:**
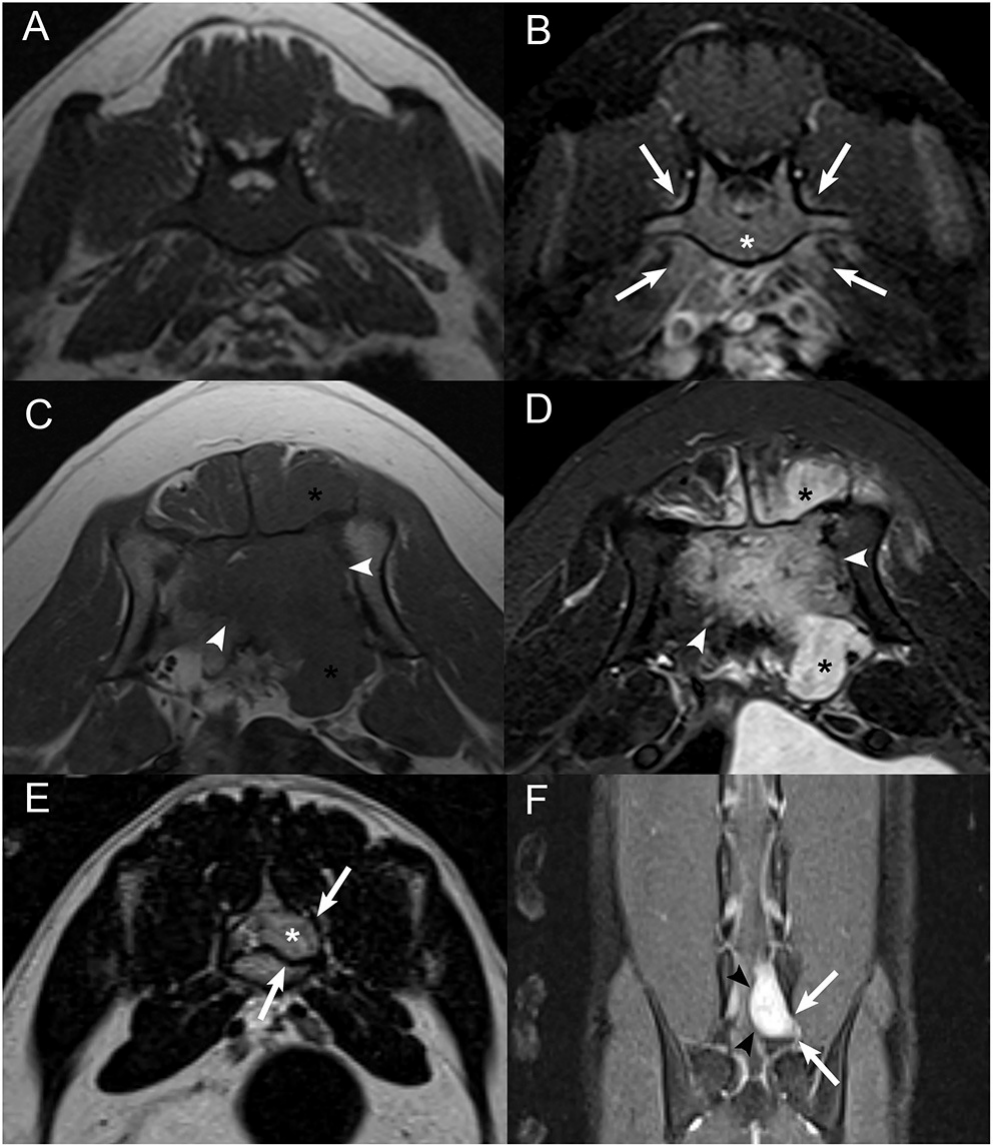
Examples of cortical sparing, cortical lysis, and benign/reactive bone change. Transverse **(A)** T1W image and **(B)** post-contrast T1W image with fat saturation at the level of the seventh lumbar vertebra in an 8-years-old male castrated Australian Cattle Dog diagnosed with lymphoma with cortical sparing of the affected bone. There is homogeneous contrast enhancement of the medullary cavity without cortical lysis (*) with mild contrast enhancement of the adjacent paraspinal soft tissues (white arrows) and contrast-enhancing soft tissue extending into the vertebral canal. Transverse **(C)** T1W image and **(D)** post-contrast T1W image with fat saturation at the level of the sacrum in an 8-years-old male castrated Great Dane diagnosed with osteosarcoma with extensive cortical lysis of the affected bone. There is a large, ill-defined, heterogeneously contrast-enhancing mass centered on the sacrum (white arrowheads) and extending into the vertebral canal and paraspinal soft tissues (*). **(E)** Transverse T2W image and **(F)** dorsal post-contrast T1W image with fat saturation at the level of the caudal aspect of L7 and L7-S1 intervertebral foramen in a 4-years-old male castrated domestic shorthair cat diagnosed with a peripheral nerve sheath tumor with benign/reactive changes of the affected bone. There is a strongly contrast-enhancing (black arrowheads) extradural mass following the L7 nerve root (*) causing smooth widening of the left aspect of the vertebral canal and of the left L7-S1 intervertebral foramen (white arrows).

In cases with bone involvement, there was a significant difference (*p* < 0.001) in the presence of cortical lysis, cortical sparing, and benign/reactive changes between all three tumor classes (Figure 1). Cortical lysis was seen in 7/7 (100%) of epithelial tumors, 17/24 (71%) of mesenchymal tumors, and 7/20 (35%) of round cell tumors. Cortical sparing was seen in 2/24 (8.3%) of mesenchymal tumors and 13/20 (65%) of round cell tumors. Benign/reactive changes were not seen in any of the epithelial tumors or round cell tumors but were seen in 5/24 (21%) of mesenchymal tumors. Tumor types associated with benign/reactive changes included three PNST, one undifferentiated sarcoma, and one soft tissue sarcoma. Notably, in the three PNSTs, these changes consisted of foraminal widening and/or pressure atrophy of the affected vertebra. Results of chi-square analysis indicated that epithelial neoplasia and mesenchymal neoplasia were more likely to have cortical lysis, whereas round cell neoplasia was more likely to have cortical sparing.

**Figure 2:**
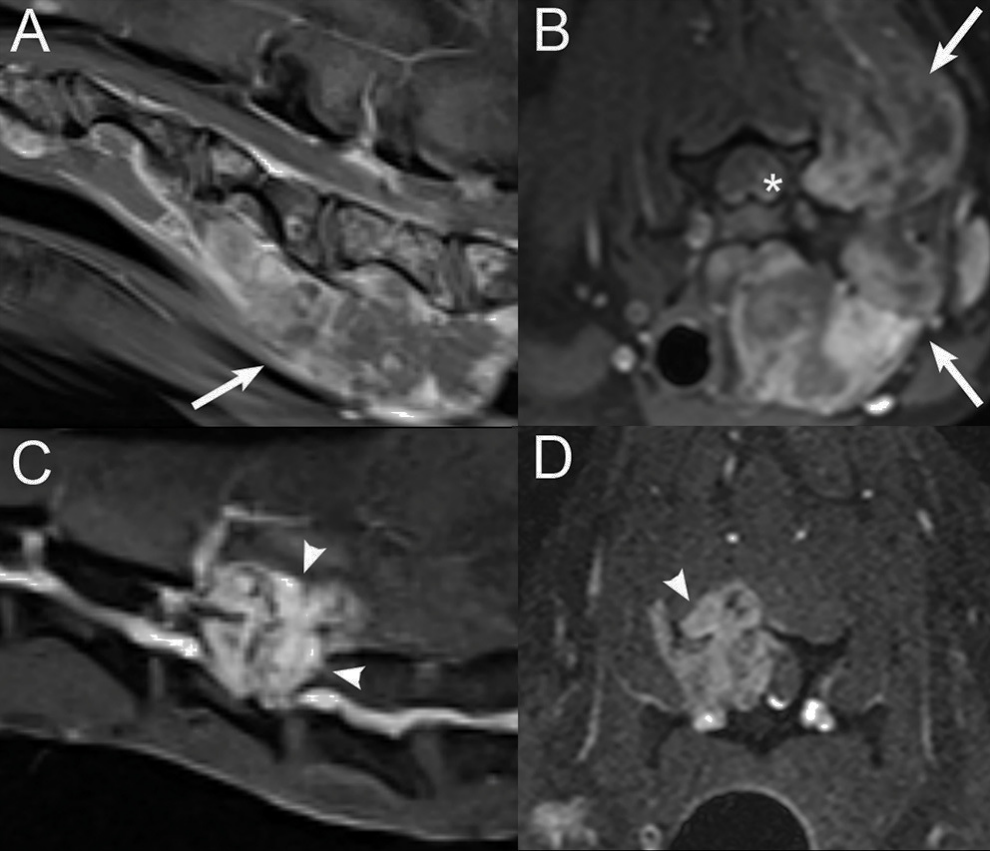
Examples of preserved vs. altered vertebral shape. Sagittal **(A)** and transverse **(B)** post-contrast T1W images with fat saturation at the level of the caudal cervical spine and sixth cervical vertebra in a 1-year-old mixed breed dog diagnosed with undifferentiated round cell neoplasia on necropsy. There is a large, ill-defined, heterogeneously contrast-enhancing mass within the paraspinal soft tissues (white arrows) and within the vertebral canal (*) along the left and ventral aspects of the vertebral canal. There is preservation of the overall shape of the vertebrae despite multifocal involvement of bone with areas of cortical lysis. Sagittal **(C)** and transverse **(D)** post-contrast T1W images with fat saturation at the level of the caudal cervical spine in a 10-years-old male castrated mixed breed dog diagnosed with hemangiosarcoma. There is a heterogeneously contrast-enhancing mass centered on the right lamina and pedicle of C5 (white arrowheads), extending into the vertebral canal and paraspinal soft tissues, with cortical and medullary lysis and with alteration of the overall shape of the vertebra.

In cases with bone involvement, chi-square analysis results indicated a significant difference in the preservation of overall shape between tumor classes (*p* = 0.008) (Figure 2). In fact, 21/26 (81%) of round cell tumors had preservation of overall shape compared with only 12/30 (40%) of mesenchymal tumors. Epithelial neoplasia did not significantly differ from the overall study population, with 5/8 (63%) having preservation of overall shape. Round cell tumors were more likely to have preservation of overall shape compared with mesenchymal tumors.

**Figure 3:**
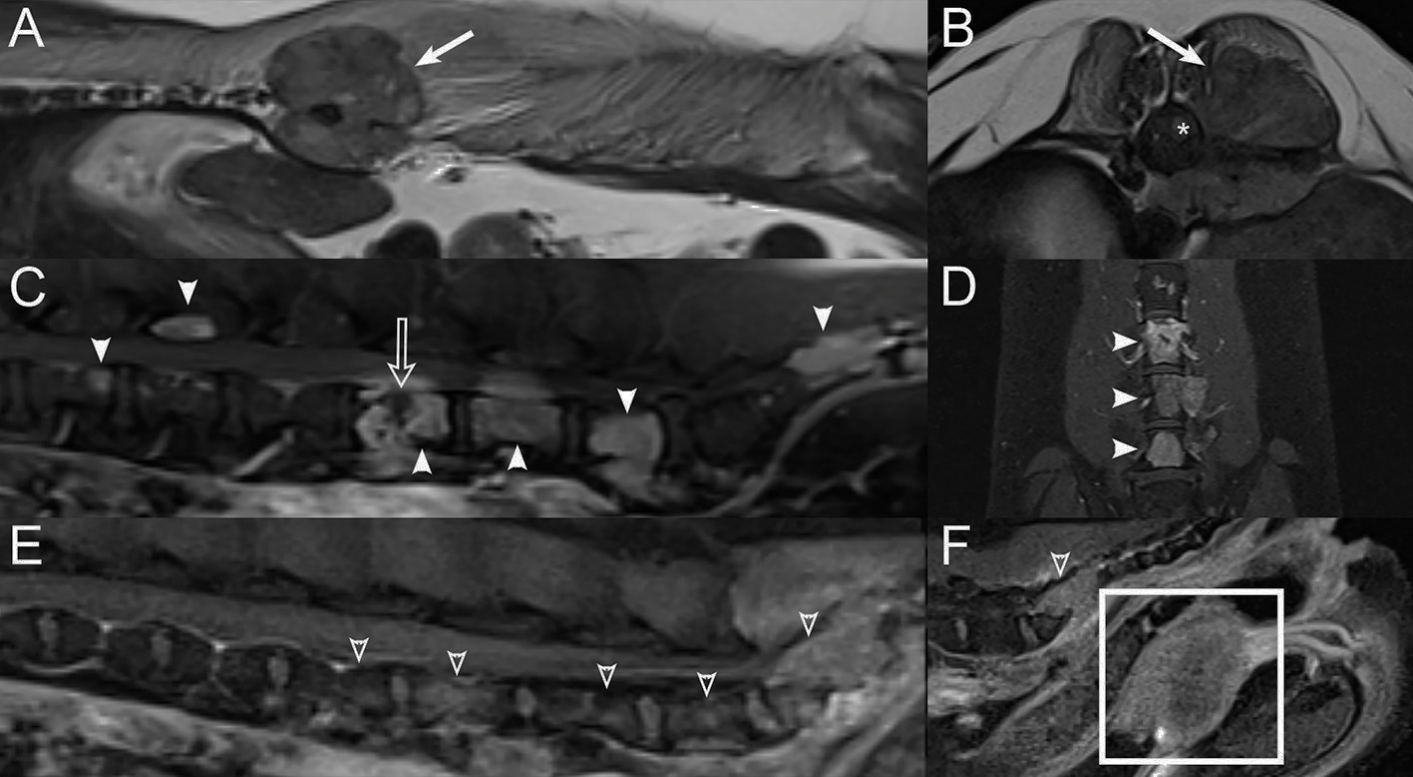
Examples of segmental vs. multifocal lesions. Sagittal **(A)** and transverse **(B)** T2W images at the thoracolumbar junction level in a 10-years-old female spayed mixed breed dog diagnosed with osteosarcoma. A segmental lesion consisting of a large soft tissue mass centered on the proximal aspect of the left 13th rib and left aspect of the 13th thoracic vertebra (white arrows) with extension into the vertebral canal (*). Sagittal **(C)** and dorsal **(D)** post-contrast T1W images with fat saturation of the caudal lumbar spine in a 10-years-old female spayed mixed breed dog diagnosed with multiple myeloma. There are multifocal contrast-enhancing vertebral lesions (white arrowheads) with a pathologic fracture (open white arrow) of the fifth lumbar vertebra. Sagittal **(E,F)** post-contrast T1W images with fat saturation of the lumbosacral spine and pelvic canal in a 6-years-old male castrated Miniature Schnauzer diagnosed with metastatic prostatic carcinoma. There are multifocal contrast-enhancing vertebral lesions (open white arrowheads). The prostate is markedly enlarged and contrast-enhancing (white rectangle).

Results of chi-square analysis indicated a significant difference (*p* = 0.008) in lesion number (segmental vs. multifocal lesion) between tumor classes (Figure 3). Mesenchymal tumors were significantly more likely to be segmental (26/30; 87%) compared with 4/30 (13%) being multifocal. In contrast, round cell neoplasia and epithelial tumors were equally likely to be segmental or multifocal. Thirteen out of 26 (50%) round cell tumors and 4/8 (50%) epithelial tumors each were segmental and multifocal, respectively.

Results of chi-square analysis indicated a significant difference in lesion centering between tumor classes (*p* = 0.004). Epithelial neoplasia was more likely to be centered on paraspinal soft tissues, with 6/8 (75%) of epithelial neoplasia centered on paraspinal soft tissues and 2/8 (25%) of epithelial neoplasia centered on bone. Round cell tumors were more likely to be centered on bone, with 16/25 (64%) of round cell tumors centered on bone and were less likely to be centered on soft tissue, with 7/25 (28%) centered on the epidural/meningeal soft tissues and 2/25 (8%) centered on paraspinal soft tissues. Mesenchymal neoplasia did not differ from the overall study population, with 12/30 (40%) centered on bone, 5/30 (17%) centered on epidural/meningeal soft tissues, and 13/30 (43%) centered on paraspinal soft tissues.

**Figure 4:**
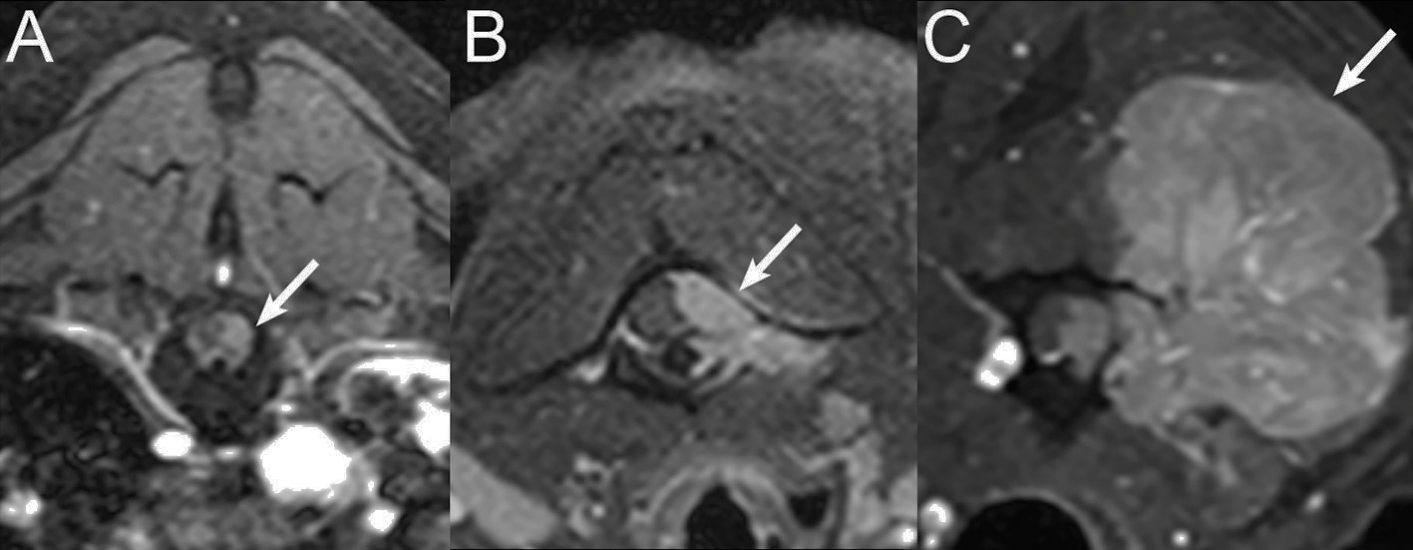
Examples of soft tissue mass size. Transverse post-contrast T1W images with fat saturation in a **(A)** 10-years-old male Labrador Retriever diagnosed with lymphoma, demonstrating small soft tissue mass size (white arrow), **(B)** 10-years-old female spayed mixed breed dog diagnosed with thyroid carcinoma with vertebral canal invasion demonstrating medium soft tissue mass size (white arrow), and **(C)** 13-years-old female spayed Golden Retriever diagnosed with undifferentiated sarcoma, demonstrating large soft tissue mass size (white arrow).

Results of chi-square analysis indicated a significant difference in soft tissue mass size between tumor classes (*p* = 0.003) (Figure 4). Round cell neoplasms were more likely to be small (18/24, 75%) than medium (2/24, 8%) or large (4/24, 17%). Mesenchymal neoplasms were more likely to be large (13/29, 45%) than medium or small (8/29, 28% each). Epithelial neoplasia was also most likely to be large (5/8, 63%).

A significant difference in contrast enhancement type (*p* = 0.029) was noted between tumor classes. Mesenchymal tumors were more likely to have heterogeneous contrast enhancement, with 27/30 (90%) displaying heterogeneous contrast enhancement. When homogeneous contrast enhancement was present, it was more likely to be seen with round cell neoplasia, with 10/26 (39%) displaying homogeneous contrast enhancement.

There was a significant difference in lesion homogeneity/heterogeneity between tumor classes on T2W sequences (*p* = 0.011). Round cell neoplasia was more likely to be homogeneous (7/26; 26.9%), whereas mesenchymal neoplasia was less likely to be homogeneous (0/30; 0%). No significant differences in lesion homogeneity/heterogeneity were seen on T1W sequences.

Results of chi-square analysis indicated a significant difference in the presence of a cavitary component to the soft tissue mass component (*p* = 0.034). Round cell tumors were less likely to be cavitary, with 0/26 (0%) of round cell neoplasia, 2/8 (25%) of epithelial neoplasms, and 6/30 (20%) of mesenchymal neoplasms having cavitary components.

A significant difference was noted in the presence/absence of meningeal enhancement between tumor classes (*p* = 0.005). Meningeal enhancement was less likely to be present with mesenchymal neoplasia, with only 3/30 (10%) having meningeal enhancement. Meningeal enhancement was seen in 4/8 (50%) of epithelial neoplasia and 12/26 (46.2%) of round cell neoplasia.

Although not significant (*p* = 0.080), results of chi-square showed a tendency for epithelial neoplasia to be polyostotic, with 6/7 (86%) epithelial tumors being polyostotic and 1/7 (14%) being monostotic. Round cell tumors had a weak tendency to be polyostotic, with 12/20 (60%) being polyostotic compared with 8/20 (40%) monostotic. Mesenchymal tumors showed a weak tendency to be monostotic, with 15/25 (60%) monostotic compared with 10/25 (40%) polyostotic.

No significant difference was seen in the invasive behavior (involvement of vertebra, vertebral canal, and paraspinal soft tissue), involvement of bone other than vertebrae, presence/absence of pathologic fractures, presence/absence of periosteal proliferation, mineralization within the soft tissue mass, degree of contrast enhancement, nerve root involvement, and spinal cord changes between classes.

**Figure 5:**
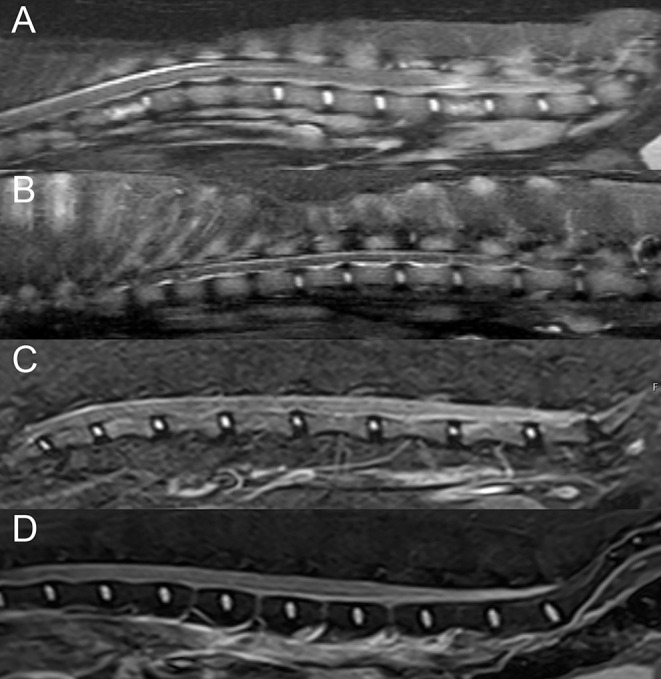
Examples of diffuse vertebral abnormalities seen with spinal lymphoma. Sagittal short-tau inversion recovery (STIR) images in a **(A)** 7-years-old male castrated Boston Terrier, **(B)** 10-years-old male castrated Australian Shepherd, and **(C)** 14-years-old male castrated domestic shorthair cat with lymphoma, demonstrating diffuse STIR hyperintensity of the included vertebrae with cortical sparing. **(D)** Sagittal STIR image in a normal 3-years-old male castrated Australian Shepherd, demonstrating normal suppression of bone marrow fat, for comparison.

Table 2 summarizes T2W and T1W signal intensities for round cell neoplasia, mesenchymal neoplasia, and epithelial neoplasia. Seven of eight (88%) of epithelial neoplasms had T1 hyperintense extradural and paraspinal components. All round cell tumors and mesenchymal tumors had T1 isointense to hyperintense extradural and paraspinal components. When bone was involved, T1-signal intensity of the osseous component was variable for epithelial and round cell neoplasia, but most mesenchymal tumors (17/22; 77.3%) had a T1-hypointense osseous component. The majority of epithelial (7/8; 87.5%), round cell (19/21; 90.5%), and mesenchymal (23/27; 85.2%) tumors had a T2-hyperintense paraspinal component. T2 signal intensity of the osseous and extradural components was variable for all three tumor classes. All but three lesions were STIR hyperintense. Two PNSTs were STIR isointense, and one calcinosis circumscripta lesion was STIR hypointense. Diffuse STIR hyperintensity of the vertebrae was noted in four patients in this study (Figure 5), three dogs and one cat with lymphoma. T2^*^ susceptibility artifacts were identified in two cases, both of which were mesenchymal neoplasms: one hemangiosarcoma and one undifferentiated sarcoma.

**Table 2:** Summary of T2-weighted and T1-weighted signal intensities for epithelial neoplasia, mesenchymal neoplasia, and round cell neoplasia in cats and dogs of this study.

	**T2 signal intensity bone**			**T2 signal intensity extradural**				**T2 signal intensity paraspinal**		
	**Hyperintense**	**Isointense**	**Hypointense**	**Hyperintense**	**Isointense**	**Hypointense**	**Mixed iso- and hypointense**	**Hyperintense**	**Isointense**	**Hypointense**
Epithelial neoplasia	4 (57.1%)	1 (14.3%)	2 (28.6%)	4 (50%)	3 (37.5%)	1 (12.5%)	0 (0%)	7 (87.5%)	0 (0%)	1 (12.5%)
Round cell neoplasia	13 (65%)	4 (20%)	3 (15%)	19 (79.2%)	5 (20.8%)	0 (0%)	0 (0%)	19 (90.5%)	2 (9.5%)	0 (0%)
Mesenchymal neoplasia	12 (54.5%)	1 (4.5%)	9 (40.9%)	17 (60.7%)	1 (3.6%)	6 (21.4%)	1 (3.6%)	23 (85.2%)	4 (14.8%)	0 (0%)
	**T1 signal intensity bone**			**T1 signal intensity extradural**				**T1 signal intensity paraspinal**		
	**Hyperintense**	**Isointense**	**Hypointense**	**Hyperintense**	**Isointense**	**Hypointense**	**Mixed iso- and hypointense**	**Hyperintense**	**Isointense**	**Hypointense**
Epithelial neoplasia	4 (57.1%)	1 (14.3%)	2 (28.6%)	7 (87.5%)	1 (12.5%)	0 (0%)	0 (0%)	7 (87.5%)	1 (12.5%)	0 (0%)
Round cell neoplasia	6 (30%)	5 (25%)	9 (45%)	9 (37.5%)	15 (62.5%)	0 (0%)	0 (0%)	12 (57.1%)	9 (42.9%)	0 (0%)
Mesenchymal neoplasia	3 (13.6%)	2 (9.1%)	17 (77.3%)	1 (3.6%)	24 (85.7%)	2 (7.1%)	1 (3.6%)	3 (11.1%)	20 (74.1%)	4 (14.8%)

Additional pertinent abdominal lesions in patients of this study included splenic nodules or masses, splenomegaly, hepatomegaly, hepatic nodules or masses, gastric wall mass, renal nodules, adrenomegaly or adrenal nodules, prostatomegaly, and bladder wall mass. Additional abdominal lesions were seen in 4/8 (50%) of patients with epithelial neoplasia, 15/26 (58%) of patients with round cell neoplasia, and 7/30 (23%) of patients with mesenchymal neoplasia. The presence of abdominal lesions was less likely in patients with mesenchymal neoplasia and more likely in patients with round cell neoplasia (*p* = 0.028). Additional pertinent thoracic lesions in patients of this study included a cranial mediastinal mass, pleural effusion, and megaesophagus with generalized fluid dilatation and were seen in 3/26 (11.5%) of patients with round cell neoplasia and no patients with mesenchymal and epithelial neoplasia. Additional lymph node lesions included lymphadenomegaly of peripheral, thoracic, or intraabdominal lymph nodes and were seen in 2/8 (25%) of patients with epithelial neoplasia, 8/26 (31%) of patients with round cell neoplasia, and 5/30 (17%) of patients with mesenchymal neoplasia. Additional pertinent musculoskeletal or soft tissue lesions included cervical masses, anal sac or perianal mass, muscle atrophy, and subcutaneous or intramuscular masses or nodules. Additional musculoskeletal or soft tissue lesions were seen in 4/8 (50%) of patients with epithelial neoplasia, 4/26 (15%) of patients with round cell neoplasia, and 4/30 (13%) of patients with mesenchymal neoplasia. No significant difference between tumor classes was seen in the presence of lymph node, thoracic, and musculoskeletal lesions.

For patients with epithelial neoplasia, 1/8 (12.5%) had mild spinal cord compression, 4/8 (50%) had moderate spinal cord compression, and 3/8 (37.5%) had severe spinal cord compression. For patients with round cell neoplasia, 2/26 (7.7%) had no spinal cord compression, 8/26 (30.8%) had moderate spinal cord compression, and 16/26 (61.5%) had severe spinal cord compression. For patients with mesenchymal neoplasia, 2/30 (6.7%) had mild spinal cord compression, 3/30 (10%) had moderate spinal cord compression, and 25/30 (83.3%) had severe spinal cord compression.

### MRI Features Based on Specific Tumor Types

A significant difference was seen (*p* = 0.027) between primary bone tumors, undifferentiated sarcomas, and PNST in the presence of cortical lysis or sparing. Six of seven primary bone tumors had cortical lysis (85.7%), and 1/7 (9.1%) had cortical sparing; 9/11 (81.8%) undifferentiated sarcomas had cortical lysis, 1/11 (9.1%) had cortical sparing, and 1/11 (9.1%) had benign/reactive changes. Of the PNSTs, 3/4 (75%) had benign/reactive change, whereas 1/4 (25%) had cortical sparing. Primary bone tumors and undifferentiated sarcomas were more likely to have cortical lysis, whereas PNSTs were more likely to have benign/reactive changes. Although there was no statistically significant difference between individual round cell tumor types for cortical lysis vs. cortical sparing, all six patients with vertebral involvement of lymphoma had cortical sparing. Three of seven patients with multiple myeloma/plasma cell tumors had cortical lysis, and 4/7 patients had cortical sparing. No significant difference was noted between individual epithelial tumor types for cortical lysis vs. cortical sparing.

A significant difference in lesion centering was noted between individual tumor types for round cell neoplasia (*p* = 0.024) and mesenchymal neoplasia (*p* = 0.008). No significant difference was noted between individual epithelial tumor types for lesion centering. Seven of seven (100%) of multiple myeloma/plasma cell tumor cases were centered on bone. Six of 12 (50%) lymphoma cases were centered on bone, whereas 6/12 (50%) were centered on the epidural soft tissues. Multiple myeloma/plasma cell tumor cases were more likely to be centered on bone, and when the lesion was centered on epidural soft tissues, it was more likely lymphoma. Eight of 10 (80%) of primary bone tumors were centered on bone, whereas 2/10 (20%) were centered on the epidural soft tissues (one hemangiosarcoma and one chondrosarcoma). Five of seven (71.4%) of PNSTs were centered on the paraspinal soft tissues, whereas 2/7 (28.6%) were centered on the epidural soft tissues. Of the undifferentiated sarcomas, 4/11 (36.4%) were centered on bone, 6/11 (54.5%) were centered on paraspinal soft tissues, and 1/11 (9.1%) were centered on epidural soft tissues. Primary bone tumors were more commonly centered on bone, whereas PNSTs and undifferentiated sarcomas were more likely to be centered on paraspinal soft tissues.

**Figure 6:**
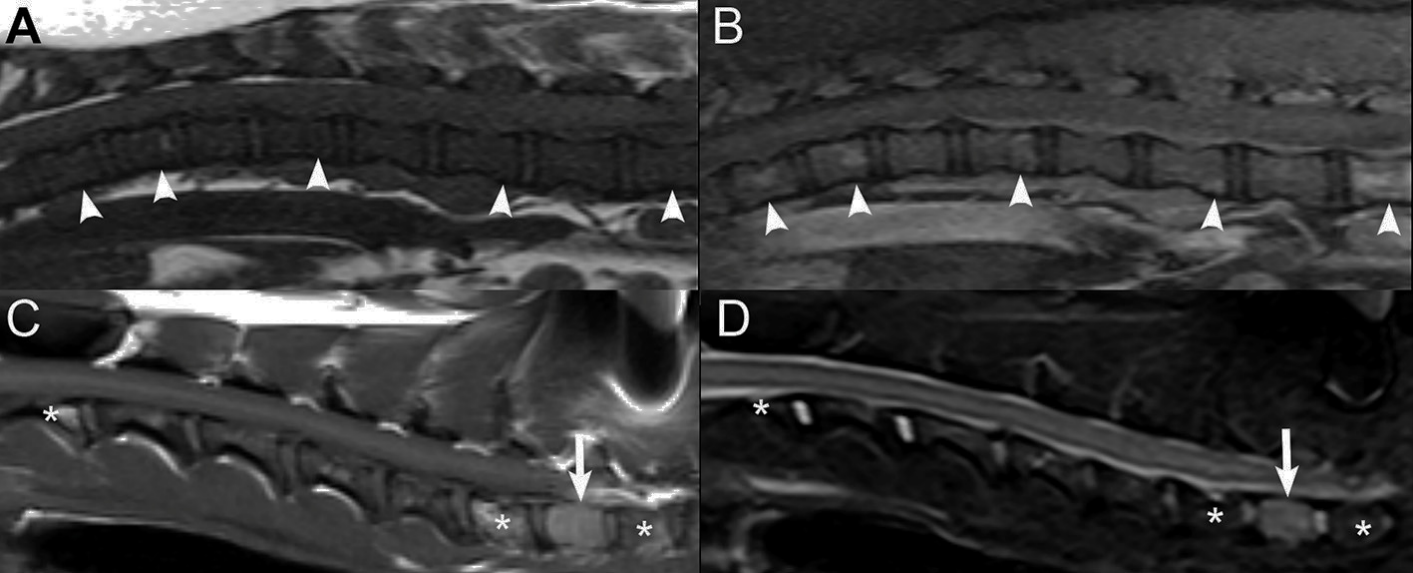
Examples of T1 signal intensity of different vertebral neoplasias. Sagittal T1W **(A)** and post-contrast T1W image with fat saturation **(B)** images in a 7-years-old male castrated Boston Terrier with lymphoma displaying T1-isointensity of the affected vertebrae (white arrowheads) and corresponding patchy heterogeneous contrast enhancement of the affected vertebrae (white arrowheads). Sagittal T1W **(C)** and STIR **(D)** images in an 11-years-old female spayed Miniature Schnauzer with a plasma cell tumor, displaying T1-hyperintensity and corresponding STIR hyperintensity of the affected first thoracic vertebra (white arrow). There are also multifocal areas of T1-hyperintensity within additional vertebrae, which suppress on the STIR sequence (*), corresponding to presumed areas of benign fatty degeneration of the bone marrow.

Table 3 summarizes the T1 and T2 signal intensities for the most commonly seen tumor types within each class. Of these, a significant difference (*p* = 0.017) was noted in T1 signal intensity of bone lesions with multiple myeloma in comparison with lymphoma, where lymphoma was more likely to be isointense, and multiple myeloma was more likely to be hyperintense (Figure 6). No additional significant differences were identified.

**Table 3:** Summary of T2 and T1-weighted signal intensities for the most commonly seen individual tumor types within the epithelial neoplasia, mesenchymal neoplasia, and round cell neoplasia tumor classes in cats and dogs of this study.

	**T2 signal intensity bone**			**T2 signal intensity extradural**				**T2 signal intensity paraspinal**		
	**Hyperintense**	**Isointense**	**Hypointense**	**Hyperintense**	**Isointense**	**Hypointense**	**Mixed iso- and hypointense**	**Hyperintense**	**Isointense**	**Hypointense**
**Epithelial neoplasia**										
Metastatic carcinoma	2 (40%)	1 (20%)	2 (40%)	2 (40%)	2 (40%)	1 (20%)		7 (77.8%)	2 (22.2%)	
Thyroid carcinoma	2 (100%)			2 (66.7%)	1 (33.3%)			5 (100%)		
**Round cell neoplasia**										
Lymphoma	2 (33.3%)	3 (50%)	1 (16.7%)	8 (72.7%)	3 (27.3%)					
Multiple myeloma	5 (71.4%)		2 (28.6%)	4 (66.7%)	2 (33.3%)			19 (90.5%)	2 (9.5%)	0 (0%)
**Mesenchymal neoplasia**										
Primary bone tumors	4 (50%)		4 (50%)	5 (71.4%)	2 (25%)			6 (75%)	2 (25%)	
PNST	1 (50%)	1 (50%)		6 (100%)				6 (100%)		
Undifferentiated sarcoma	6 (54.5%)		5 (45.5%)	9 (81.8%)	2 (18.2%)			9 (81.8%)	2 (18.2%)	
	**T1 signal intensity bone**			**T1 signal intensity extradural**				**T1 signal intensity paraspinal**		
	**Hyperintense**	**Isointense**	**Hypointense**	**Hyperintense**	**Isointense**	**Hypointense**	**Mixed iso- and hypointense**	**Hyperintense**	**Isointense**	**Hypointense**
**Epithelial neoplasia**										
Metastatic carcinoma	2 (40%)	1 (20%)	2 (40%)	4 (80%)	1 (20%)			4 (80%)	1 (20%)	
Thyroid carcinoma	2 (100%)			3 (100%)				3 (100%)		
**Round cell neoplasia**										
Lymphoma		4 (66.7%)	2 (33.3%)	4 (36.4%)	7 (63.6%)	0 (0%)	0 (0%)	3 (33.3%)	6 (66.7%)	
Multiple myeloma	4 (57.1%)		3 (42.9%)	4 (66.7%)		2 (33.3%)		4 (80%)	1 (20%)	
**Mesenchymal neoplasia**										
Primary bone tumors	1 (12.5%)		7 (87.5%)		6 (75%)	1 (12.5%)	1 (12.5%)	1 (12.5%)	5 (62.5%)	2 (25%)
PNST	1 (50%)	1 (50%)			7 (100%)			2 (33.3%)	4 (66.6%)	
Undifferentiated sarcoma	1 (9.1%)	1 (9.1%)	9 (81.8%)	1 (9.1%)	9 (81.8%)	1 (9.1%)			9 (81.8%)	2 (18.2%)

A significant difference (*p* = 0.001) was noted in the presence of nerve root involvement between mesenchymal tumor types. Nerve root involvement was seen in 6/7 (85.7%) of PNSTs. None (0/10; 0%) of the primary bone tumors had nerve root involvement. Nerve root involvement was seen in 7/11 (63.6%) of undifferentiated sarcomas. PNST was more likely to have nerve root involvement.

No significant difference was noted between individual tumor types for round cell neoplasia, epithelial neoplasia, and mesenchymal neoplasia for segmental vs. multifocal lesions, monostotic vs. polyostotic lesions, preservation of overall shape, soft tissue mass size, lesion homogeneity/heterogeneity on T2W and T1W sequences, contrast enhancement type, and degree of contrast enhancement.

### Binary Logistic Regression

Binary logistic regression was performed to determine if certain features or combinations of features could predict tumor class between round cell neoplasia and mesenchymal neoplasia. Logistic regression analysis using the combination of features preservation of overall shape, contrast enhancement type, and lesion centering was able to correctly classify a lesion into mesenchymal neoplasia or round cell neoplasia 78.2% of the time, accurately predicting 84% of round cell neoplasia and 73% of mesenchymal neoplasia. The largest contributor was the feature preservation of overall shape. If the overall shape was not preserved, the lesion was 10.2 times more likely to be mesenchymal neoplasia. Additionally, if the contrast enhancement type was heterogeneous, the lesion was 5.8 times more likely to be mesenchymal neoplasia. Finally, if the lesion was centered on paraspinal soft tissues, it was 9.0 times more likely to be mesenchymal neoplasia than if it was centered on bone. If the lesion was centered on the epidural soft tissues, there was no discernible difference between round cell neoplasia and mesenchymal neoplasia.

## Discussion

In dogs, the most common extradural tumor classes in this study were mesenchymal tumors, followed by round cell neoplasia, and epithelial neoplasia, with the most common tumor types being undifferentiated sarcoma, nerve sheath tumor, osteosarcoma, lymphoma, multiple myeloma, and metastatic carcinoma. Similarly, in a retrospective study of 394 primary and secondary spinal tumors, mesenchymal tumors were also the most common tumor class, occurring in 69.1% of cases, whereas round cell neoplasia was less common than reported here, occurring only in 8.9% of cases (29). Tumors from hematopoietic and fatty marrow elements of bone had previously been described as being exceedingly rare in dogs in a radiographic study of primary and secondary spinal tumors (31). It is possible that with the increasing use of MRI, these neoplasms are being identified with increasing frequency due to the ability of MRI to detect changes in bone marrow signal intensity, which may not be visible radiographically. Carcinomas were the most common extradural metastatic tumors in the present study, in agreement with previous reports (2).

As expected, in cats, round cell neoplasia was the most common tumor class, with infrequent mesenchymal and epithelial tumors. In all cats diagnosed with round cell neoplasia, lymphoma was the diagnosed tumor type. This is consistent with previous studies, where lymphoma was reported to be the most common spinal cord tumor in cats and the second most common spinal cord disease, with the first being feline infectious peritonitis (28, 30). Feline lymphoma has been reported to have a bimodal age distribution, affecting cats younger than 3 years of age, usually associated with feline leukemia virus infection, and also affecting cats older than 8 years of age, which usually are feline leukemia virus-negative (40). This is consistent with the wide age distribution of cats affected by spinal lymphoma in this study, with ages ranging 1 and 20 years of age, with a median age of 14 years.

As expected, there was an overlap in MRI features; however, certain features were more likely to be seen with certain tumor classes. Given the low numbers of each individual tumor type within each tumor class, statistical analysis was inconclusive for most evaluated MRI characteristics; however, a few MRI features were significant even between individual tumor types within each class.

The individual MRI features more likely to be seen with round cell neoplasia were cortical sparing, preservation of overall shape, lesion centering on bone, small soft tissue tumor size, and homogeneous contrast enhancement. All patients with vertebral involvement of lymphoma in this study had cortical sparing, whereas patients with multiple myeloma/plasma cell tumor had a relatively even distribution of cortical lysis and cortical sparing. The finding of cortical sparing has been previously reported in patients with vertebral lymphoma; however, other reports have described areas of multifocal cortical lysis with lymphoma (33, 41–43). In a recent study evaluating the MRI features of dogs with multiple myeloma, most patients had expansile lesions that remained largely confined within cortical boundaries (37). The vertebral bone involvement with lymphoma and multiple myeloma in multiple dogs of this report, whether it be associated with cortical lysis or cortical sparing, is likely a reflection of the frequent bone marrow involvement with these disease processes (33, 37). The finding of cortical lysis in some patients with multiple myeloma in this study may be a reflection of MRI performed at a later stage of the disease process. Despite the finding of cortical lysis in some cases of round cell neoplasia in this study, the overall shape of the vertebra was more likely to be preserved with round cell neoplasia. This is consistent with a previous MRI report in a patient with polyostotic lymphoma describing multifocal areas of cortical bone lysis but with the maintenance of a high percentage of the osseous anatomy (23).

Additionally, within the round cell neoplasia tumor class, multiple myeloma was more likely to be centered on bone. This is not surprising given that multiple myeloma has a predilection for the bone marrow of the axial skeleton (37). When the lesion was centered on the epidural soft tissues, it was more likely lymphoma. In fact, a soft tissue mass centered on the epidural soft tissues without evidence of bone involvement was seen in 3/7 cases of lymphoma in dogs of the present report. This is consistent with a previous report of spinal lymphoma, where one dog had evidence of neoplastic tissue within the vertebral canal without osseous involvement (33). A similar pattern of solitary epidural lymphoma has also been reported in people (44).

**Figure 7:**
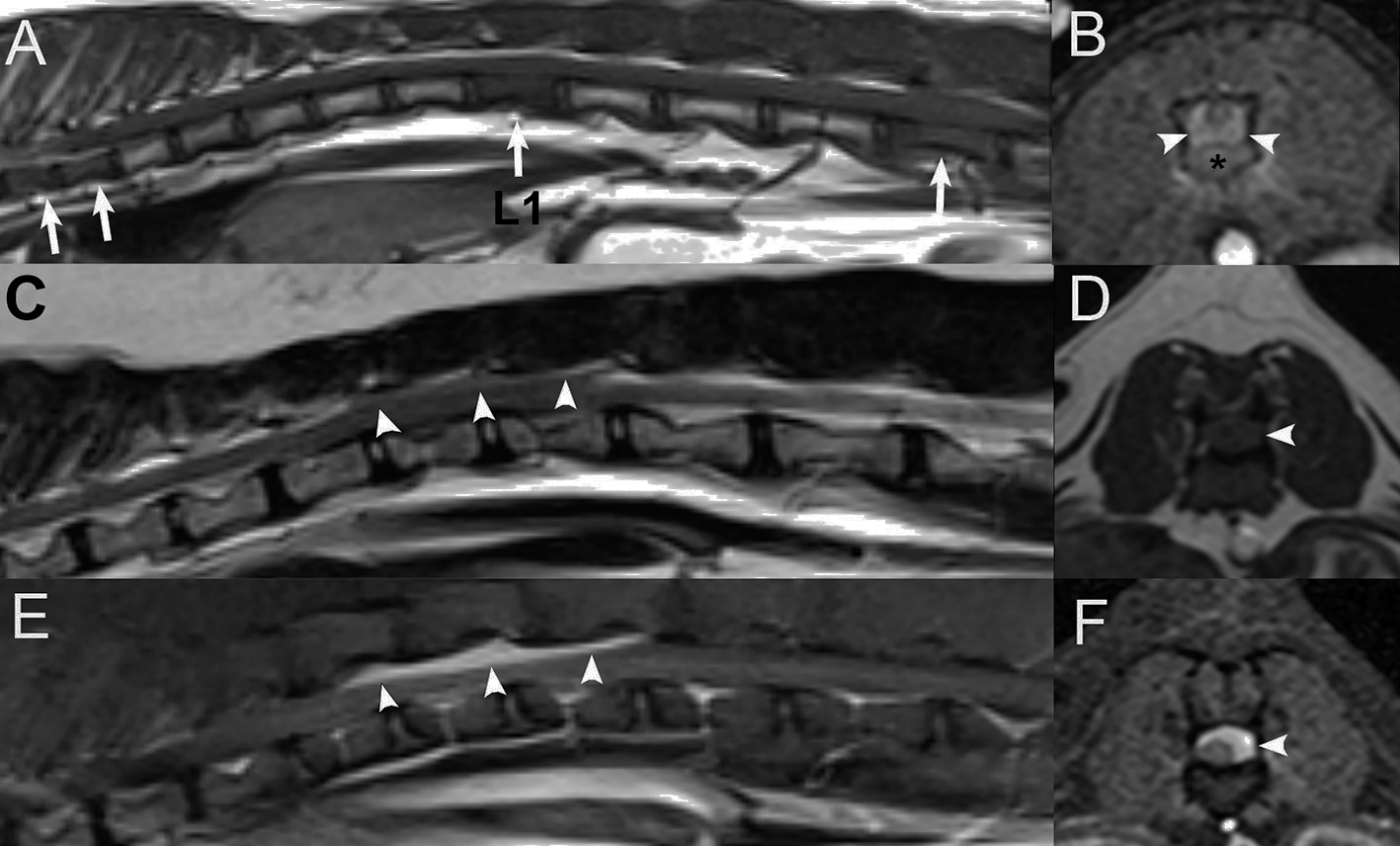
Examples of different presentations of feline spinal lymphoma. Sagittal T1W image **(A)** and transverse post-contrast T1W image with fat saturation at the level of L1 **(B)** in a 1-year-old male castrated domestic shorthair cat displaying multifocal vertebral T1-hypointensity at T7, T8, L1, and L5 (white arrows) with a contrast-enhancing epidural mass at the level of L1 (white arrowheads) with contrast-enhancement of the vertebra (*) and paraspinal soft tissues. **(C)** Sagittal T2W, **(D)** transverse T1W, and sagittal **(E)** and transverse **(F)** post-contrast T1W images with fat saturation displaying a T2-mildly hyperintense, T1-isointense, contrast-enhancing epidural mass extending from T12 to L1 (white arrowheads) in a 14-years-old male castrated domestic shorthair cat without bone involvement.

In cats diagnosed with lymphoma, as expected, MRI findings were similar to those seen in dogs, where some cats had polyostotic bone lesions with cortical sparing, with multifocal sites of extradural spinal cord compression, whereas others had lesions centered on the epidural soft tissues with no evidence of bone involvement (Figure 7). For this reason, feline and canine lymphoma cases were grouped for statistical analysis. The MRI findings in cats with lymphoma in the current report are similar to what has previously been described in cats with central nervous system lymphoma, notably, epidural lesions with or without the involvement of vertebral bodies or skeletal muscle (34, 40). Finally, spinal cord lymphoma has been reported to have a predilection for the lumbosacral and thoracic tracts in cats (30, 34). Of the five cats with spinal lymphoma in this study, two cats had lesions at the level of the L4 to S3 vertebrae, one had lesions at the level of the T3-L3 vertebrae, and two cats had lesions at the level of the T3 to L3 and L4 to S3 vertebrae, which is consistent with previous reports. However, none of the cats in this study had an MRI of the cervical spine, so additional cervical lesions cannot be entirely excluded.

The T1 and T2 signal intensities of lesions from all classes were highly variable, and T1 hyperintensity was seen with tumors of all classes. Despite the overlap between tumor classes and types, within the round cell neoplasia tumor class, bone lesions with multiple myeloma were more commonly T1-hyperintense, whereas bone lesions with lymphoma were more commonly T1 isointense. This is consistent with previous reports in dogs describing lymphoma as hypointense to isointense on T1W sequences and multiple myeloma as hyperintense on T1W sequences (33, 37). However, a previous study in cats has described lymphoma as being slightly hyperintense to gray matter on T1W images (45). Additionally, although the T1-signal intensity of the osseous component was variable for epithelial and round cell neoplasia, most mesenchymal tumors (77.3%) had a T1-hypointense osseous component. This is consistent with the previously reported iso- or hypointensity on T1W imaging in dogs with osteosarcoma and chondrosarcoma (26, 46).

Diffuse STIR hyperintensity of the vertebrae was noted in four patients with round cell neoplasia in this study, all four of which were lymphoma. Failure of bone marrow suppression on STIR sequences has been reported with spinal lymphoma and has been attributed to the replacement of normal bone marrow fat by malignant neoplastic cells, inflammation, and/or necrotic tissue (33). Most of these lesions were T1 and T2 isointense and, therefore, were most conspicuous on STIR sequences, which is similar to results of a previous study on spinal lymphoma (33).

The individual MRI features more likely to be seen with mesenchymal neoplasia were cortical lysis, segmental lesion, heterogeneous contrast enhancement, a cavitary component to the soft tissue mass, and medium or large size of the mass. Heterogeneous contrast enhancement has been described with some mesenchymal tumors, such as chondrosarcoma and osteosarcoma, although there is variability in the reported contrast-enhancement type of other mesenchymal tumors, with primary vertebral hemangiosarcoma and extradural hemangiosarcoma displaying strong homogeneous contrast enhancement and osteosarcoma in a cat reported to display homogeneous contrast enhancement (26, 45–48). Nonetheless, 90% of patients with mesenchymal neoplasia in the present report had heterogeneous contrast enhancement. When heterogeneous contrast enhancement is noted in a medium to large extradural spinal mass, mesenchymal neoplasia may be more likely.

As expected, within the mesenchymal neoplasia tumor class, primary bone tumors (chondrosarcoma, osteosarcoma, and hemangiosarcoma) were more commonly centered on bone, whereas nerve sheath tumors and undifferentiated sarcomas were more likely to be centered on the paraspinal soft tissues. Peripheral nerve sheath tumors can grow and extend from an extradural location to an intradural-extramedullary location, and eventually, may be intramedullary (2). Of the six included peripheral nerve sheath tumors of the present report, two extended into an intradural-extramedullary location, and none were intramedullary. It is therefore not surprising that lesion centering for these tumors was most commonly the paraspinal soft tissues. Furthermore, as expected, nerve root involvement was more common with nerve sheath tumors. Therefore, an extradural mass centered on a nerve root should raise suspicion for a peripheral nerve sheath tumor, even when not in the more classic intradural-extramedullary location.

Primary bone tumors and undifferentiated sarcomas were more likely to have cortical lysis, whereas nerve sheath tumors were more likely to have benign/reactive changes. This is in agreement with a previous study evaluating the radiographic features of primary and secondary spinal tumors, where the major radiographic change affecting the majority of primary bone tumors was lysis, although, in that study, primary vertebral tumors presented with both productive and destructive features (31). Both lytic and sclerotic areas were described in a previous study evaluating the MRI features of osteosarcoma (1). Furthermore, a recent study evaluating the MRI features of vertebral chondrosarcomas described the lesions as osteolytic and proliferative masses (46). Although primary osseous hemangiosarcoma is rare, a recent case report described the MRI findings of a primary osseous hemangiosarcoma, where there was an invasion of the L2 spinous process, with an extension of neoplastic tissue into the vertebral canal, and with an expansion of the L2 vertebra upon visual inspection (47). Hemangiosarcoma was seen in two patients of the present report. In one of these patients, this lesion had features of a primary bone tumor similar to the case report previously mentioned, as it was centered on bone, and had cortical lysis with extension into the vertebral canal (47). However, in the second patient, the lesion consisted of a primary extradural mass adhered to the dura mater with invasion of the leptomeninges, without osseous involvement. In this patient, T2^*^ susceptibility artifacts were also identified, which likely represented intralesional hemorrhage, which was not unexpected considering the tumor type. Hemangiosarcoma has previously been reported to occur as a primary extradural mass, similar to one of the patients of this report (48). Finally, with nerve sheath tumors, affected foramina can become enlarged secondary to bone resorption due to nerve enlargement, which is consistent with the benign osseous changes seen more commonly with nerve sheath tumors in patients of the present report (49).

**Figure 8:**
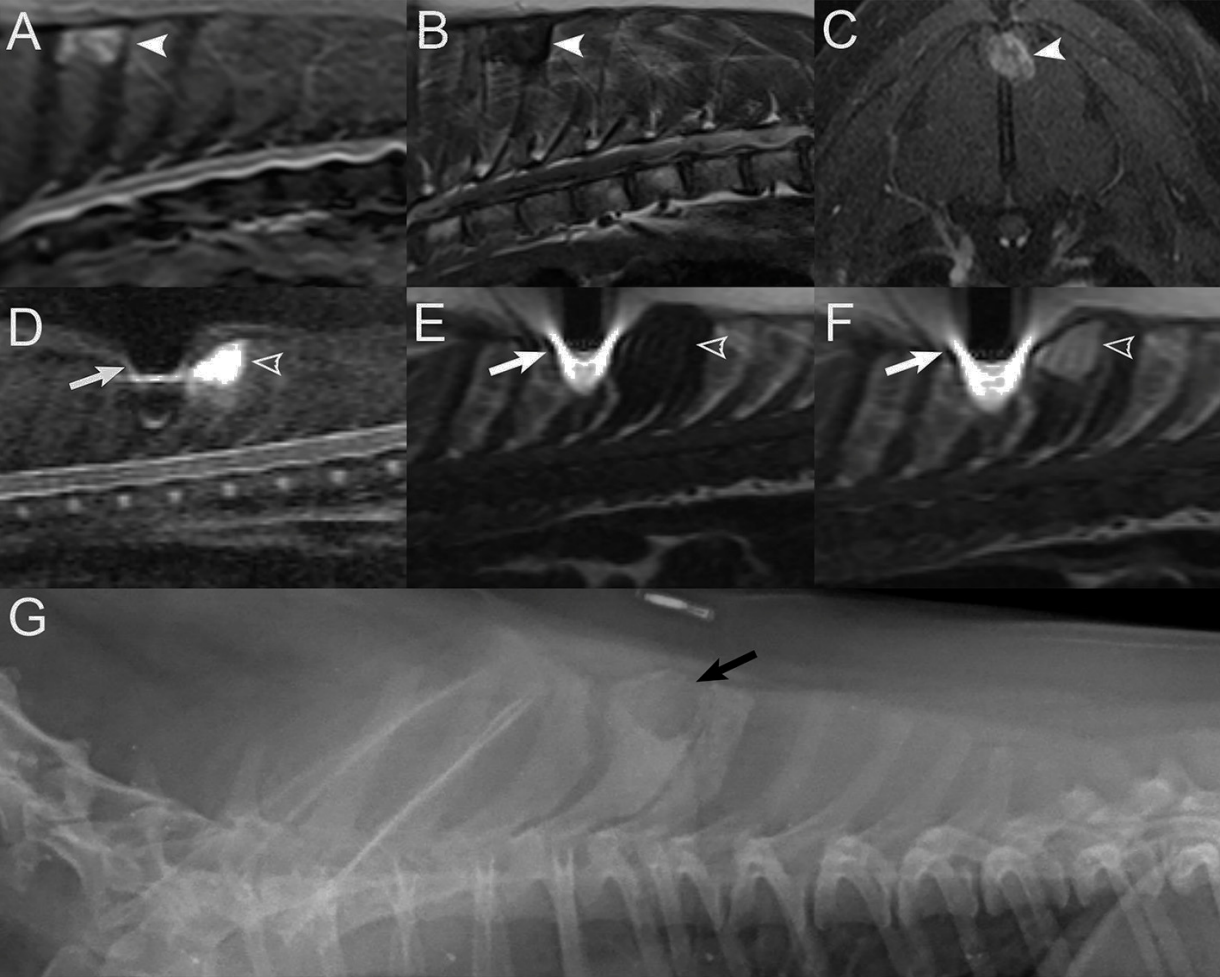
Examples of imaging findings with vertebral chondrosarcoma. Sagittal STIR **(A)** and T1W **(B)** images and transverse post-contrast T1W image with fat saturation **(C)** in a 12-years-old female spayed mixed breed dog displaying a STIR-hyperintense, T1-hypointense, and heterogeneously contrast-enhancing mass centered on the spinous process of T4 (white arrowheads). Sagittal STIR **(D)**, T1W **(E)**, and post-contrast T1W with fat saturation **(F)** images in a 5-years-old male castrated Maltese displaying a STIR-hyperintense, T1-hypointense, and moderately contrast-enhancing mass centered on the dorsal aspect of the spinous process of T4 (open white arrowheads). There is a large signal void caused by the patient's subcutaneous identification microchip (white arrows). **(G)** Lateral radiograph of the dog in **(D–F)** displaying a well-defined region of geographic expansile lysis surrounded by sclerosis of the dorsal aspect of the spinous process T4 (black arrow) with mild irregular lysis and surrounding sclerosis of the caudal aspect of the spinous process of T3 and patchy sclerosis of the spinous process of T5.

Two of three dogs of this study with chondrosarcoma had lesions centered on the dorsal aspect of their respective spinous process, with T1 and T2 hypointensity of the spinous processes immediately cranial and caudal to the principal lesion in one of these cases (Figure 8). Similarly, in a recent study evaluating the MRI features of vertebral chondrosarcoma, all six cases had lesions involving the dorsal compartments (spinous process lamina and apophyses) of several consecutive vertebrae (46). This is in contrast to what has been described for typical primary vertebral neoplasms, which usually affect single vertebrae, most often at the level of the vertebral body (31, 46). Similar findings have been described in humans, where up to 85% of chondrosarcomas have been reported to affect the posterior elements of the vertebrae (46). This has been hypothesized to be due to the presence of three growth centers within the vertebrae from which tumors can arise (46, 50). The third chondrosarcoma in our study consisted of an ovoid, heterogeneously contrast-enhancing, extradural mass in the lumbar vertebral canal without osseous involvement. Primary extradural chondrosarcoma with similar imaging features has previously been reported in the cervical spine of a 10-years-old Labrador Retriever, although in this case, it was associated with focal thinning of the adjacent vertebra (51).

The individual MRI features more likely to be seen with epithelial neoplasia were cortical lysis, lesion centering on paraspinal soft tissues, a cavitary component to the soft tissue mass, and medium or large mass size.

**Figure 9:**
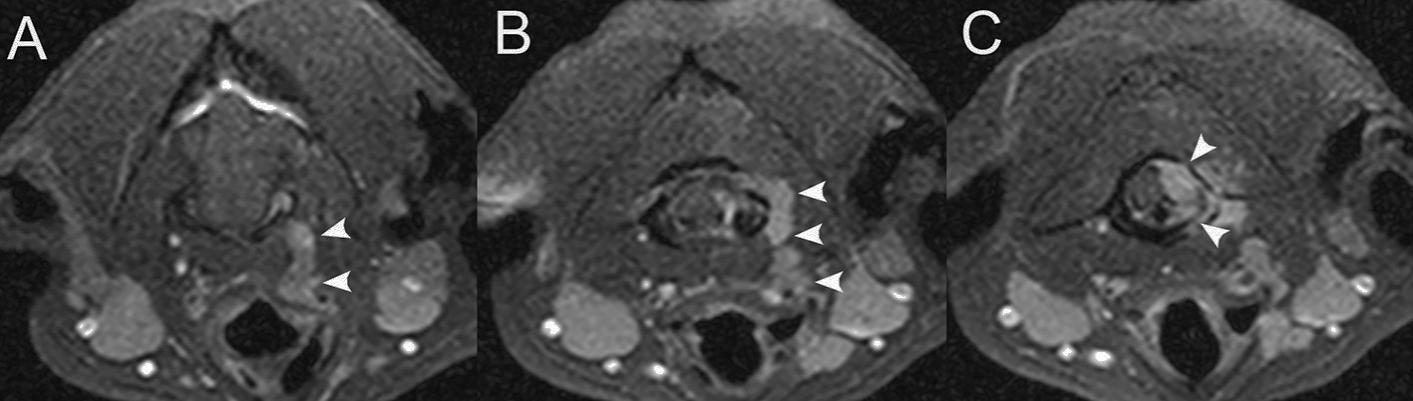
Thyroid carcinoma extension into the vertebral canal. Sequential transverse T1W post-contrast images with fat saturation at the atlantooccipital junction level, from rostral to caudal **(A–C)** in a 10-years-old female spayed mixed breed dog with a history of incompletely resected thyroid carcinoma. A serpentine, markedly homogeneously contrast-enhancing left ventral cervical mass is present in the region of the left thyroid lobe, extending dorsally and caudally into the vertebral canal (white arrowheads), resulting in moderate extradural spinal cord compression.

The majority (5/8) of epithelial neoplasms were metastatic tumors secondary to various primary neoplasms, which included prostatic carcinoma, hepatic cholangiocarcinoma, apocrine gland adenocarcinoma of the anal sac, and intestinal carcinoma. This is not surprising, given that carcinomas have been reported to be one of the most common tumor types associated with extradural metastases (2). Carcinomas of mammary, lung, prostatic, and urinary bladder origin are the most commonly reported tumors associated with spinal metastases (52, 53). Apocrine gland adenocarcinoma of the anal sac has also uncommonly been reported to metastasize to the spine (53, 54). Interestingly, three of seven epithelial tumors were thyroid carcinoma with invasion of the spine and intracranial invasion through regions of lysis of the skull and the cranial cervical vertebrae (Figure 9). Previous reports describe epidural metastases of thyroid carcinoma resulting in spinal cord compression, but to the authors' knowledge, this is the first report of direct extension of the primary tumor into the vertebral canal with associated bone lysis and spinal cord compression (55, 56). This behavior has also been previously described with carotid body tumors (or paragangliomas) and was also seen in one patient of the present report with a carotid body tumor (57). Thyroid carcinoma and carotid body tumors should be considered in cases of cervical masses with intracranial and/or cervical vertebral canal invasion.

Although not significant, there was a tendency for epithelial neoplasia and round cell neoplasia to be polyostotic and mesenchymal neoplasia to be monostotic, which is consistent with the expected behavior of these tumor classes. There was no significant difference in this study between tumor class or tumor type and the number of involved compartments (paraspinal soft tissues, vertebral canal, and vertebrae), and the majority of spinal tumors involved more than one compartment. Previous studies have described the involvement of more than one spinal compartment in patients with lymphoma (33). Case reports in dogs with vertebral osteosarcoma, chondrosarcoma, and hemangiosarcoma also describe the involvement of two or more spinal compartments (36, 46, 47). Finally, no difference was seen between the degree of spinal cord compression between tumor classes, with all tumor classes most commonly causing moderate or severe spinal cord compression. Therefore, the number of affected vertebral compartments or degree of spinal cord compression were not features that helped differentiate between tumor classes.

When combining the MRI features preservation of overall shape, type of contrast enhancement, and lesion centering, it was possible to correctly classify a lesion as mesenchymal neoplasia or round cell neoplasia with 78.2% accuracy, predicting 84% of round cell neoplasia and 73% of mesenchymal neoplasia. The combination of features predictive of round cell neoplasia was the preservation of overall shape, homogeneous contrast enhancement, and lesion centering on bone, whereas alteration of overall shape, heterogeneous contrast enhancement, and lesion centering on paraspinal soft tissues were predictive of mesenchymal neoplasia. Additional prospective studies are required to further evaluate the accuracy of these feature combinations in diagnosing classes of extradural spinal tumors.

**Figure 10:**
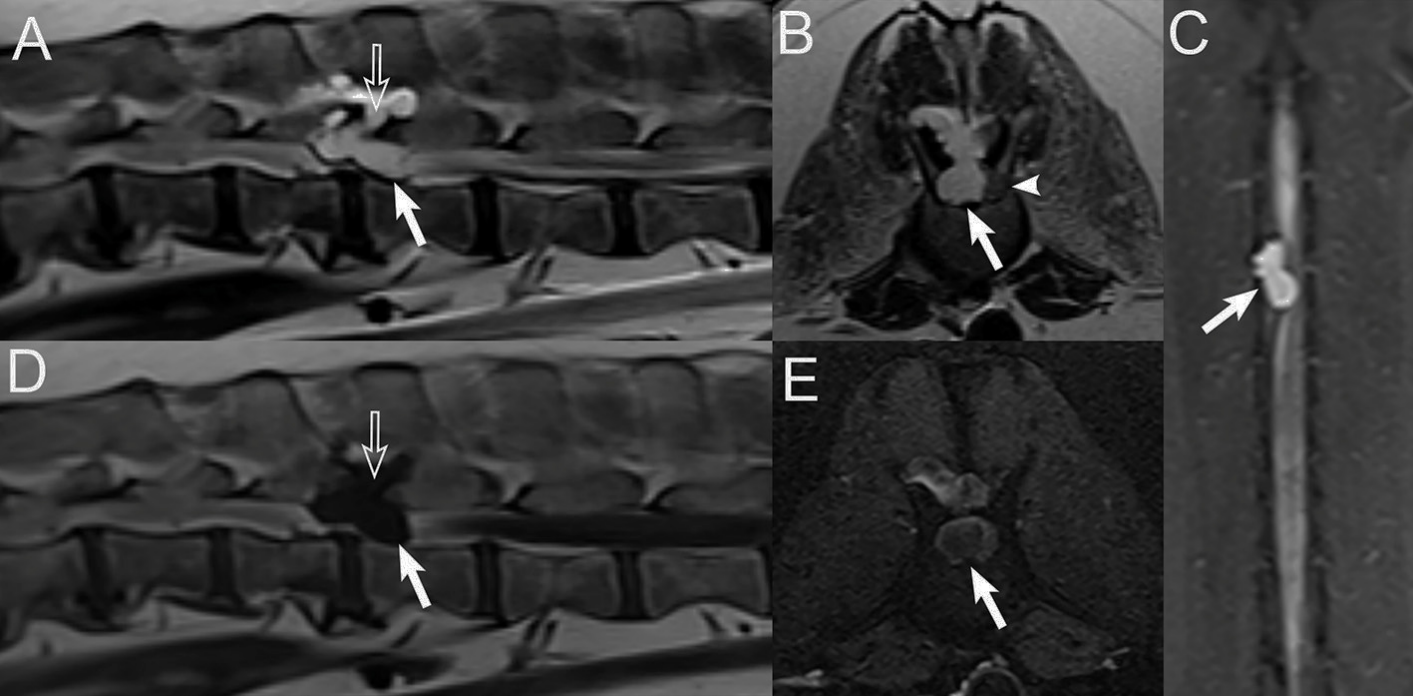
Synovial myxoma. Sagittal **(A)** and transverse **(B)** T2W images, dorsal STIR image **(C)**, sagittal T1W image **(D)**, and transverse T1W post-contrast image with fat saturation **(E)** in an 11-years-old male castrated Labrador Retriever. A T2-strongly hyperintense, T1-hypointense lobulated cystic mass with mild peripheral rim enhancement (white arrows) is centered on the right L1-L2 articular process joint, which is widened (open white arrows). This mass extends into the vertebral canal and is associated with severe extradural spinal cord compression and leftward deviation (white arrowhead).

**Figure 11:**
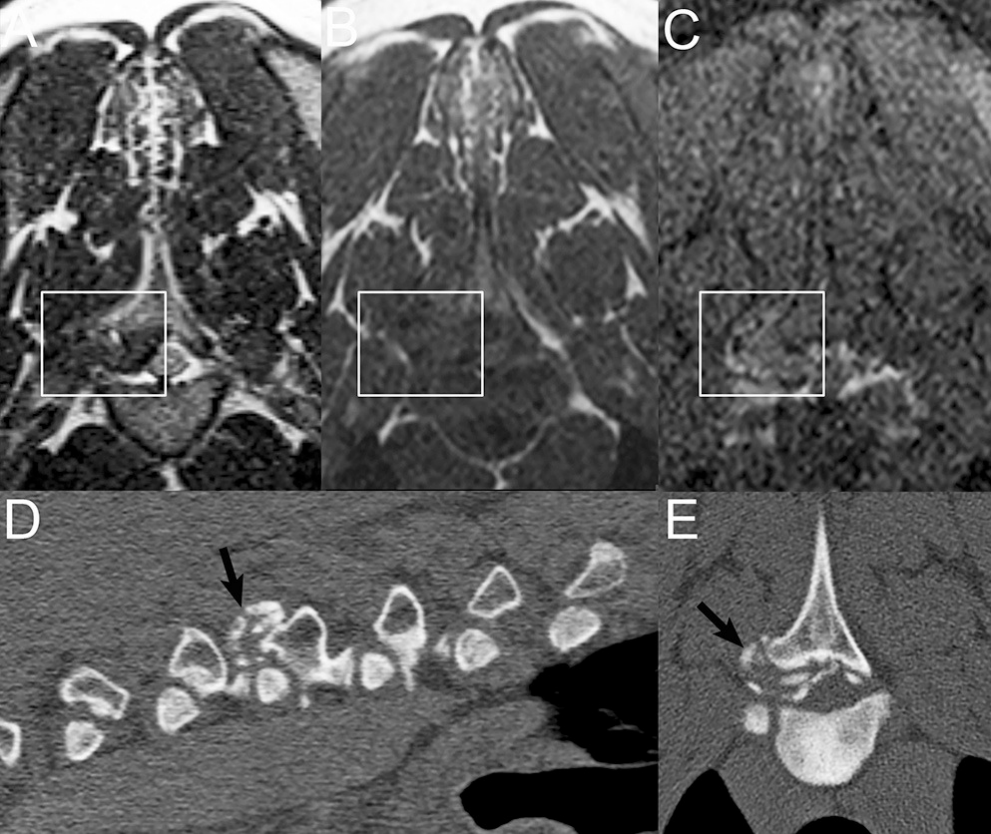
Calcinosis circumscripta. Transverse T2W **(A)**, T1W **(B)**, and T1W post-contrast with fat saturation **(C)** images in a 1-year-old male German Shepherd. There is an ill-defined T2-heterogeneously hypointense, T1-hypointense, and mildly heterogeneously contrast-enhancing proliferative lesion centered at the right T3-T4 articular process joint (white rectangle), protruding into the vertebral canal and resulting in moderate extradural spinal cord compression. Corresponding sagittal multiplanar reconstructed **(D)** and transverse **(E)** CT images demonstrating an ill-defined stippled mineralized mass-lesion centered at the right T3-T4 articular process joint (black arrows) and protruding into the vertebral canal.

Similar to what has been previously reported, benign vertebral neoplasms and benign proliferation of bone affecting the spine were uncommon in our study population, with one dog having a benign joint-associated neoplasm, a synovial myxoma, and one dog having calcinosis circumscripta (2). Vertebral synovial myxomas have been uncommonly reported in the veterinary literature, with only four case reports identified to the authors' knowledge (16, 17, 58, 59). MRI features have been described in two case reports and are similar to what was seen in the patient of the present report, which was a multiloculated T2 hyperintense, T1 hypointense mass with moderate peripheral contrast enhancement, centered on an articular process joint and resulting in severe extradural spinal cord compression (Figure 10). Calcinosis circumscripta has been previously described in the cervical spine of a dog, occurring concurrently with multiple cartilaginous exostoses, and was thought to occur in response to the disruption of surrounding tissue by the multiple cartilaginous exostoses (22). Calcinosis circumscripta is defined as ectopic mineralization of soft tissues, usually adjacent to, or involving periarticular structures, and usually occurs in young, large-breed dogs (22). In the patient of the present report, calcinosis circumscripta was the sole lesion and was T1 and T2 heterogeneously hypointense, mildly heterogeneously contrast-enhancing and was associated with moderate extradural spinal cord compression (Figure 11).

### Limitations

There were several limitations to the present report, with the first being its retrospective design. Cases without a cytologic or histopathologic diagnosis were not included in the present report, which may have introduced a selection bias, with the exclusion of more severely affected patients, such as in cases of pathologic fractures. Histopathology was not available for all patients, and special staining allowing further identification of a specific tumor type was often not performed. Therefore, in many cases, further narrowing the diagnosis from a tumor class into a specific tumor type was not possible. This led to low numbers within each specific tumor type, with many round cell tumors and mesenchymal tumors being subcategorized into round cell tumor of undetermined origin and undifferentiated mesenchymal tumor. This likely limited statistical evaluation of possible differences between individual tumor types within each tumor class. A sampling of the tumor was not performed in all cases, and in some cases, the diagnosis was indirectly reached by sampling lesions in other organs (and presumed to be the same etiology in the vertebra).

For statistical analysis, due to the low numbers of each tumor type, it was chosen to combine osteosarcoma, chondrosarcoma, and hemangiosarcoma into a group named primary bone tumors. Although one of the included hemangiosarcoma cases had features consistent with a primary bone tumor, the second case was a primary extradural hemangiosarcoma without bone involvement. Similarly, one case of chondrosarcoma in the present report also presented as an extradural mass without osseous involvement. Therefore, the inclusion of hemangiosarcoma and chondrosarcoma into this subgroup may have minimally skewed results of statistical analysis for primary bone tumors. Another limitation was using the most aggressive feature for statistical analysis in some cases of polyostotic bone involvement that had a mix of cortical sparing and cortical lysis. Of these, some cases were primarily cortical sparing, with only one or a few sites of cortical lysis. This would likely impact the prioritization of differential diagnoses when evaluating the case individually. Because the entire spine was not imaged in all patients, the determination of monostotic vs. polyostotic lesions may be skewed in some cases. There was a lack of standardization between imaging protocols, which were adapted to the motive for imaging and the neurolocalization, and image quality varied between studies. This might have influenced statistical analysis results if some sequences were not performed or available for certain patients. Finally, approximately half of the studies were performed using a 1T magnet and half using a 1.5-T magnet, likely resulting in variations in lesion signal intensity and homogeneity. A prospective study using a larger population and a standardized imaging protocol could be considered in the future to remedy this.

## Conclusion

In conclusion, despite some overlap in imaging features between extradural spinal neoplasia classes and types, certain imaging features may help prioritize differential diagnoses. When considering combined features, preservation of overall shape, homogeneous contrast enhancement, and lesion centering on bone were able to accurately predict 84% of round cell neoplasia, whereas the combined features alteration of overall shape, heterogeneous contrast enhancement, and lesion centering on paraspinal soft tissues were able to accurately predict 73% of mesenchymal neoplasia. In addition, certain individual MRI features such as cortical sparing, preservation of overall shape, lesion centering on bone, small soft tissue tumor size, and homogeneous contrast enhancement were more likely to be seen with round cell neoplasia. Cortical lysis, a cavitary component to the soft tissue mass, and medium to large soft tissue mass size were more likely to be seen with mesenchymal neoplasia and epithelial neoplasia, although cortical lysis did occur in some cases of round cell neoplasia in the present report. Chondrosarcoma should be considered as a differential diagnosis in cases of tumors centered on the dorsal vertebral compartments, as was the case for two of the three chondrosarcomas of the present report. In cases of well-defined, contrast-enhancing, ovoid extradural masses in dogs, consideration should be given to chondrosarcoma or hemangiosarcoma, with the latter being prioritized if intralesional T2^*^ susceptibility artifacts are present. In cases of cervical masses with calvarial and cervical vertebral canal invasion, thyroid carcinoma and carotid body tumors should be considered. Finally, a few cases of round cell neoplasia, in particular, lymphoma, had diffuse STIR hyperintensity of the vertebrae, which may have been missed without the inclusion of a fat-suppressed sequence. In agreement with previous studies, a STIR sequence, either in the sagittal or dorsal plane, should be part of the imaging protocol of the spine, especially if round cell neoplasia is a consideration (33, 37). Although findings from the present report can aid in prioritizing differential diagnoses, tissue sampling remains the gold standard for definitive diagnosis. Further larger prospective studies would be required to further evaluate the findings of the present report.

## Data Availability Statement

The raw data supporting the conclusions of this article will be made available by the authors, without undue reservation.

## Author Contributions

MA and SH: conception, design, acquisition of data, and drafting the article. MA, SH, and CS: analysis and interpretation of data, revising article for intellectual content, and final approval of the completed article. All authors contributed to the article and approved the submitted version.

## Conflict of Interest

The authors declare that the research was conducted in the absence of any commercial or financial relationships that could be construed as a potential conflict of interest.
